# Discovery and Characterization of IFITM *S*-Palmitoylation

**DOI:** 10.3390/v15122329

**Published:** 2023-11-28

**Authors:** Tandrila Das, Howard C. Hang

**Affiliations:** 1 Immunology Program, Memorial Sloan Kettering Cancer Center, New York, NY 10065, USA; 2 Departments of Immunology and Microbiology and Chemistry, Scripps Research, La Jolla, CA 92037, USA

**Keywords:** IFITM3, *S*-palmitoylation, influenza A virus

## Abstract

Interferon-induced transmembrane proteins (IFITM1, 2 and 3) are important host antiviral defense factors. They are active against viruses like the influenza A virus (IAV), dengue virus (DENV), Ebola virus (EBOV), Zika virus (ZIKV) and severe acute respiratory syndrome coronavirus (SARS-CoV). In this review, we focus on IFITM3 *S*-palmitoylation, a reversible lipid modification, and describe its role in modulating IFITM3 antiviral activity. Our laboratory discovered *S*-palmitoylation of IFITMs using chemical proteomics and demonstrated the importance of highly conserved fatty acid-modified Cys residues in IFITM3 antiviral activity. Further studies showed that site-specific *S*-palmitoylation at Cys72 is important for IFITM3 trafficking to restricted viruses (IAV and EBOV) and membrane–sterol interactions. Thus, site-specific lipid modification of IFITM3 directly regulates its antiviral activity, cellular trafficking, and membrane-lipid interactions.

## 1. Introduction

**Figure 1:**
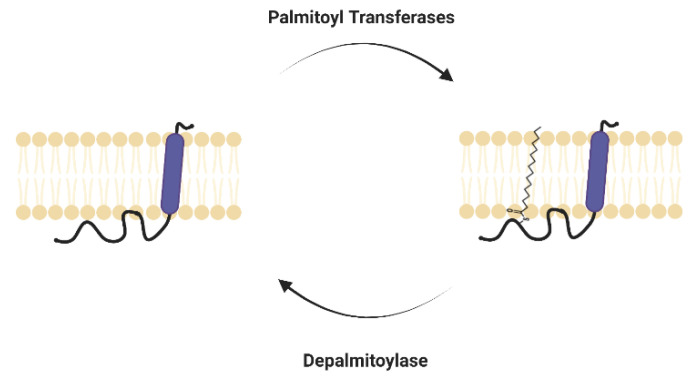
*S*-palmitoylation of proteins. Dynamic *S*-palmitoylation is mediated by DHHC palmitoyl acyltransferases (DHHC-PATs) and depalmitoylases.

The post-translational modification of proteins plays an important role in regulating protein structure and function. *S*-palmitoylation is a post-translational modification of proteins with lipids. It typically involves the addition of a 16-carbon-long palmitic acid to cysteine residues of a protein (Figure 1) [1]. The high-energy thioester bond between the fatty acyl group and cysteine residue results in *S*-palmitoylation being a unique, reversible lipid modification [2].

The role of *S*-palmitoylation is implicated in protein trafficking, targeting different membrane compartments, lipid microdomains, protein stability, conformation, and protein interactions [3]. For example, *S*-palmitoylation of soluble H- and N-ras enables their compartmentalization between the plasma membrane and the Golgi apparatus, and therefore the diversification of signal transduction [4]. Reported *S*-palmitoylated proteins include enzymes, receptors, ion channels, transporters, innate immunity effectors, and many others. Overall, *S*-palmitoylation regulates a diverse array of physiological processes, including cellular signaling, transcriptional regulation and others [5].

Palmitoyl acyltransferases (PATs) mediate the *S*-palmitoylation of proteins [6]. There are 23 PAT family proteins in humans. They have an Asp-His-His-Cys (DHHC) domain essential for catalysis. DHHC-PATs were first discovered in yeast and are conserved across all eukaryotes. The localization, substrate profiles and functionality of individual enzymes are still not very well understood. The removal of palmitate from proteins, i.e., the hydrolysis of thioesters, is regulated by depalmitoylases [7]. Acyl-protein thioesterase 1 (APT1) and acyl-protein thioesterase 2 (APT2) were first identified as depalmitoylases for G proteins. Additionally, α/β-hydrolase domain-containing protein 17 (ABHD17) and other serine hydrolase superfamily proteins were also identified as depalmitoylases.

In this review, we outline the discovery and role of IFITM3 *S*-palmitoylation and describe the different chemical biology tools that have been developed and used to study IFITM3 *S*-palmitoylation, sites of modification and the role of modification in vitro and in cells. Here, we also aim to provide a mechanistic understanding of *S*-palmitoylation-regulated IFITM3’s trafficking in cells, membrane–sterol interactions, specificity, and activity against viral infections.

### 1.1. Discovery of IFITM3 S-Palmitoylation

The interferon-induced IFITM1, IFITM2 and IFITM3 are palmitoylated at Cys [8,9]. IFITM3 Cys is highly conserved across most mammals and is required for the antiviral activity of IFITM3 orthologues from mice, bats, and humans [10,11]. IFITM3 *S*-palmitoylation, sites of modification and their role in regulation of IFITM3 activity have been identified using integrated chemical biology, cell biology and biochemical approaches.

**Figure 2:**
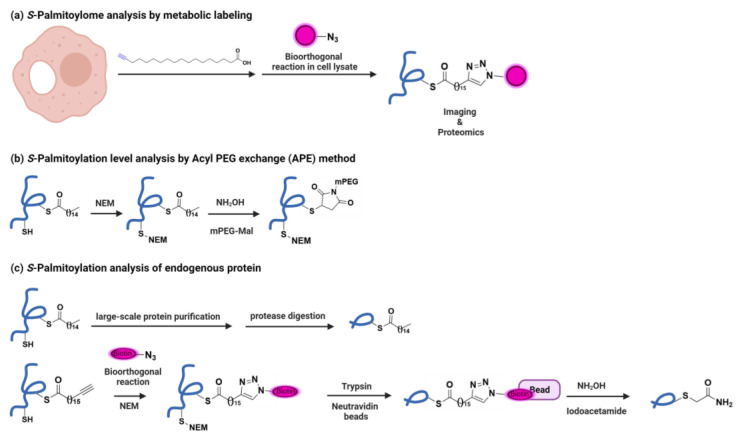
Discovery and analysis of IFITM3 *S*-palmitoylation. (**a**) Metabolic labeling of cells with palmitic acid reporters and further reaction with azido-modified fluorescent or affinity tags (in pink) for fluorescent visualization or affinity enrichment of *S*-palmitoylated proteins enabled discovery of IFITM3 *S*-Palmitoylation. (**b**) Acyl-PEG exchange (APE) involves the capping of free cysteine residues with N-ethyl maleimide (NEM) and the removal of fatty acid groups with hydroxylamine (NH_2_OH). Then, the exposed cysteines are reacted with mPEG-Mal. Proteins are separated via SDS-PAGE and analyzed using Western blot, enabling the detection of both unmodified and *S*-fatty acylated proteins. (**c**) *S*-palmitoylation analysis of endogenous proteins. Also, palmitic acid reporter modified proteins are reacted with azide-biotin (in pink) and captured on Neutravidin beads. Then, thioesters are hydrolyzed with hydroxylamine (NH_2_OH) and alkylated with iodoacetamide before LC-MS/MS analysis. Cys modified by iodoacetamide are marked as S-fatty-acylation sites.

IFITM3 *S*-palmitoylation was discovered by means of fatty acylation profiling of mouse dendritic cells [9]. Fatty acylation profiling uses palmitic acid chemical reporters, i.e., palmitic acid-like molecules containing azido or alkynyl groups, to label cysteines of target proteins using endogenous palmitoylation machinery (Figure 2a). Using bioorthogonal ligation methods, they can be reacted with biotin for the enrichment and identification of palmitoylated proteins via proteomics. They can also be reacted with azide-fluorophore for gel-based imaging. Palmitic acid reporter labeling of overexpressed IFITM1, 2 and 3 confirmed their palmitoylation. Site-directed mutagenesis identified IFITM3 *S*-palmitoylation at three Cys residues (Cys71, C72 and 105) and its importance in IFITM3 antiviral activity against the influenza A virus.

As a complementary chemical approach, acyl-PEG exchange (APE) enables the identification of IFITM3 *S*-palmitoylation levels in cells [8]. APE involves the modification of thioester-linked cysteines in *S*-palmitoylated proteins (Figure 2b). The sensitivity of thioesters to hydroxylamine (NH_2_OH) has also previously been explored to selectively capture *S*-acylated proteins in acyl–biotin exchange (ABE) or acyl–resin capture (acyl-RAC), but APE readily reveals the fraction of unmodified versus *S*-fatty acylated proteins or the number of sites of *S*-acylation [12,13]. In APE, free cysteines of palmitoylated proteins are capped and thioester linkages are subsequently cleaved to generate new thiols. These thiols are then selectively labeled using PEG as a mass tag for mobility-shift-based assays to identify levels of protein *S*-palmitoylation. APE analysis of single Cys to Ala mutant IFITM3 showed that Cys72 is the major site of modification of IFITM3. This was also confirmed by means of metabolic labeling of IFITM3 Cys mutant with palmitic acid chemical reporter. Cys72 is also the major site of modification for IFITM1 and IFITM2.

Further, a method based on fatty acylation enrichment and selective cleavage of thioester bonds with hydroxylamine led to *S*-fatty-acylation site identification of endogenous IFITM3 (Figure 2c) [14]. Here, *S*-fatty-acylated proteins are metabolically labeled with alk-16 and are biotinylated with azido-biotin. Free Cys are capped with N-ethyl maleimide (NEM), proteins are digested, and biotinylated proteins are enriched on Neutravidin beads. Then, thioesters are selectively hydrolyzed with hydroxylamine and alkylated again with iodoacetamide before LC-MS/MS analysis. Cys modified with iodoacetamide are marked as *S*-fatty-acylation sites. This confirmed IFITM3 Cys71 and 72 as sites of lipid modification. Also, mass spectrometric analysis of purified IFITM3 from mammalian cells revealed Cys72 as the predominant site of *S*-palmitoylation.

Thus, the development and use of these chemical tools helped in the discovery of IFITM3 *S*-palmitoylation and in identifying sites of modification and the most important Cys for *S*-palmitoylation. The use of such chemical reporters and bioorthogonal labeling for characterizing various biological pathways has been extensively reviewed [15,16].

### 1.2. DHHC-PATs and Depalmitoylases of IFITM3

Substrate profiles of individual DHHC-PATs are regulated at the transcriptional, translational and post-translational levels [17]. Initial screening of cell lines individually lacking DHHC1-24 showed that the knockout of single PAT does not inhibit IFITM3 *S*-palmitoylation or antiviral activity, suggesting functional redundancy among PATs. Overexpression studies with the 23 DHHCs show that multiple palmitoyltransferases (DHHC 3, 7, 15, and 20) are involved in IFITM3 *S*-palmitoylation, but DHHC20 is the most important for IFITM3 antiviral activity [18]. DHHC20 also co-localized with IFITM3 in lysosomes, whereas DHHC 3, 7 and 15 showed perinuclear localization, suggesting the location of palmitoylation might influence activity.

Recently, α/-hydrolase domain-containing 16A (ABHD16A) was identified as a depalmitoylase for IFITMs. Depalmitoylase activity of ABHD16A was conserved across humans, mice and pigs. ABHD16A was identified as interacting with IFITMs using the yeast two-hybrid (Y2H) assay and the protein coimmunoprecipitation (co-IP) assay. Knockdown of ABHD16A leads to the accumulation of IFITMs in the plasma membrane, suggesting its role in IFITM trafficking from the plasma membrane to the cytoplasm and the antiviral activity of IFITMs [19]. Stable expression of ABHD16A inhibited Japanese encephalitis virus (JEV) infection. Moreover, a specific inhibitor of ABHD16A increased the susceptibility of cells to JEV infection [19]. Knockout of ABHD16A in HEK293T also implicated the role of ABHD16A in inhibiting the entry of thrombocytopenia syndrome virus (SFTSV) and vesicular stomatitis virus (VSV) pseudotypes in cells [19].

### 1.3. S-Palmitoylation and Other PTMs

IFITMs, which are small type IV single-pass transmembrane proteins, are highly regulated by post-translational modifications (PTMs) [20,21,22,23]. In addition to *S*-palmitoylation at membrane-juxtaposed Cys residues (Cys71, 72 and 105), Tyr20 phosphorylation regulates IFITM3 plasma membrane localization and endocytosis, whereas Lys ubiquitination at residues 24, 83, 88, and 104, and especially at Lys24, is important for IFITM3 trafficking and turnover in cells [9,24,25,26]. Lys 88 is also monomethylated by SET7 and it negatively regulates IFITM3 antiviral activity. Even though individual PTMs have been widely studied, how each of these modifications works in concert with other co- and post-translational modifications to regulate protein functions is yet to explored in detail. These PTMs also regulate IFITM3 interactions with membrane lipids and proteins as discussed in a later section [27]. 

## 2. IFITM3 *S*-Palmitoylation and Trafficking

Live cell trafficking studies of IFITM3 during virus infection led to a significant understanding of the mechanism of IFITM3 antiviral activity and the role of site-specific *S*-palmitoylation [28,29]. However, live cell imaging has been elusive, as N- and C-terminal fusion of IFITM3 with fluorescent proteins (GFP and mCherry) results in the aggregation in lysosomes and loss of function. Suddala et al. developed IFITM3-iEGFP, IFITM3 with EGFP inserted in the N-terminal cytoplasmic region of IFITM3, whereas Peng et al. established a method combining genetic code expansion with bio-orthogonal reactions for IFITM3 labeling and imaging in live cells [29,30]. Spence et al. further used this method developed in the Hang lab to study the role of site-specific palmitoylation on IFITM3 trafficking [28].

**Figure 3:**
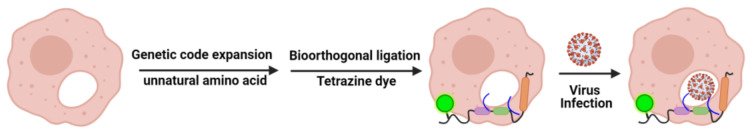
Genetic code expansion enables the site-specific incorporation of an unnatural amino acid into a protein utilizing cellular translation machinery, which further enables bio-orthogonal ligation with tetrazine dyes for live cell labeling and imaging studies.

Genetic code expansion enables the generation of proteins with unnatural amino acids that contain functional groups that can be subsequently labeled with reactive fluorogenic dye using bioorthogonal chemistry [31]. Genetic code expansion uses the cellular protein synthesis machinery to generate proteins with unnatural amino acids incorporated site-specifically. It involves using an orthogonal aminoacyl-tRNA synthetase/tRNA pair to incorporate the desired unnatural amino acid at a specific site on the protein of interest generally in response to the amber stop codon (UAG) on mRNA. Thus, genetic code expansion needs minimal perturbation of the protein of interest for fluorophore tagging. Among the different bioorthogonal reactions, inverse-electron-demand Diels–Alder cycloaddition (IEDAC) between strained alkynes or alkenes with tetrazines, a fast bioorthogonal live cell compatible reaction, has emerged as the reaction of choice for labeling proteins in live cells. Thus, a comparison of genetic code expansion and bio-orthogonal chemistry is a fast method to specifically ligate a small fluorescent dye to IFITM3 in live cells (Figure 3).

To understand the role of IFITM3 during infection and interactions of IFITM3 with virus particles, live cell imaging of fluorescently labeled IFITM3 is performed in synchronized labeled influenza A virus-infected cells. The influenza A virus was labeled with membrane-incorporated dye (DiD), which undergoes fluorescent dequenching upon hemifusion or fusion between viral and endosomal membranes. IFITM3 colocalized with the majority of internalized virus particles prior to lipid mixing. These results show that while virus particles may fuse with IFITM3-positive vesicles, IFITM3 does not significantly inhibit virus hemifusion with endocytic membranes. To further investigate the role of *S*-palmitoylation in IFITM3 trafficking to virus particles, single Cys mutants of IFITM3 were analyzed. The trafficking and colocalization of IFITM3 Cys72 to Ala mutants with dequenched DiD-virus particles were significantly decreased compared to WT IFITM3 or Cys71 and Cys105 to Ala mutants. In comparison to WT IFITM3, Cys72 to Ala mutant trafficking to DiD-virus particles was also delayed. While *S*-palmitoylation of IFITM3 does not affect the protein levels or cellular distribution of the protein, site-specific *S*-palmitoylation of IFITM3 at Cys72 is important for its rate of trafficking to influenza A particles. Thus, Cys72 *S*-palmitoylation is important for IFITM3 trafficking and localization to virus containing vesicles.

## 3. Site-Specific Palmitoylation and Membrane Interactions

**Figure 4:**
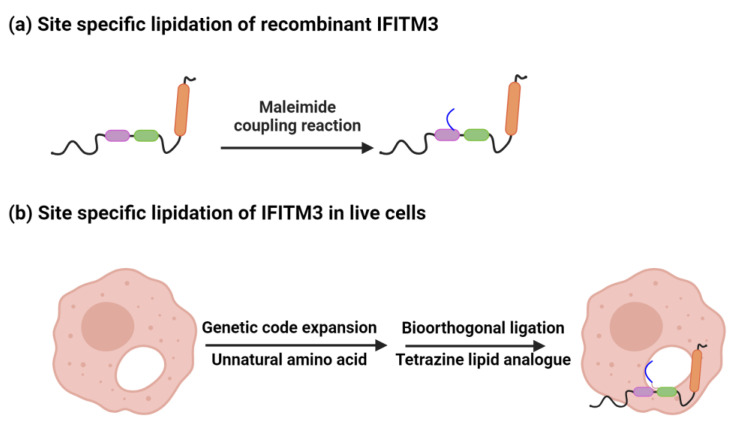
Chemical lipidation of recombinant IFITM3 and IFITM3 in cells. (**a**) Site-specific lipidation of recombinant IFITM3 using maleimide coupling. (**b**) Scheme for the site-specific lipidation of IFITM3 via genetic code expansion for unnatural amino acid incorporation and the bio-orthogonal tetrazine ligation reaction).

Multiple studies identified Cys72 as the major site of *S*-palmitoylation, and further loss-of-function studies showed that the mutagenesis of Cys72 severely impacts the trafficking and antiviral activity of IFITM3. However, the enhancement of IFITM3 activity via controlled, site-specific lipidation has not been explored. In general, S-fatty acylation remains a challenging and underexplored modification when compared with other post-translational modifications, because of physical properties such as hydrophobicity and reversibility. But new developments in chemical biology have allowed for the study of site-specific S-fatty acylation in vitro and in living cells [32]. Biophysical and structural studies with recombinant IFITM3 chemically modified at Cys72 provided further insights into the role of site-specific *S*-palmitoylation. Garst et al. generated recombinant IFITM3 with Cys71 and Cys105 mutated to Ala. They modified IFITM3 Cys72 with maleimide-palmitate (Figure 4a) and showed that lipid modification at Cys72 stabilizes IFITM3 amphipathic helix membrane interaction, which is important for the restriction of virus infections [20,33]. They performed a classical floatation assay with IFITM3^1−106^, containing amphipathic helices but lacking transmembrane region, in liposomes. Upon lipidation at Cys72, this construct showed significantly more association with liposomes. Moreover, solution-state NMR studies with full-length IFITM3 showed that the modification might have both a local and a distal effect on the protein secondary structure or membrane environment, as made evident by the changes in the α-helical propensities of residues close to the modification and also residues further away from the modification but closer to the transmembrane domain. Furthermore, molecular dynamic simulations also supported the stabilization of the functionally significant amphipathic region when lipidated at Cys72. On the other hand, genetic code expansion and bio-orthogonal chemistry with tetrazine-lipid derivatives were employed to install a stable *S*-palmitoylation mimic in live cells (Figure 4b). Chemically lipidated IFITM3 shows endolysosomal localization like endogenous IFITM3 and restores antiviral activity in comparison to a Cys72 to Ala loss-of-function mutant. Thus, the live-cell chemical lipidation approach provided the first evidence for a gain of function via site-specific lipidation of IFITM3 in mammalian cells. It also shows the importance of Cys72 *S*-palmitoylation in IFITM3 antiviral activity. Thus, in silico and in vitro studies with recombinant IFITM3 and in-cell studies with chemically lipidated IFITM3 illustrated the importance of site-specific palmitoylation at Cys72 in the regulation of IFITM3’s structure, membrane interactions and antiviral activity.

## 4. *S*-Palmitoylation, Lipid Interactions and Antiviral Specificity of IFITMs

*S*-palmitoylated IFITM3 interacts with other host proteins and membrane lipids. For example, proteomic analysis of the *S*-palmitoylated IFITM3 interactome identified many membrane-associated protein interaction partners, including the p97/VCP ATPase, which contributes to IFITM3 lysosomal turnover and antiviral activity [24,34,35]. Furthermore, yeast two-hybrid screening revealed IFITM3’s interaction with vesicle-membrane-protein-associated protein A (VAPA), which has been implicated in cholesterol accumulation in endolysosomal compartments and IFITM3’s antiviral activity [36,37]. However, other studies indicate that high cholesterol accumulation in these compartments via U18666A pretreatment or down-regulation of Niemann-Pick C1 (NPC1) does not inhibit IAV hemagglutinin-mediated viral membrane fusion [38]. On the other hand, it has been suggested that the presence of cholesterol in model membranes can promote IFITM3 amphipathic helix-mediated modulation of membrane curvature and stiffness to block fusion pore formation [20,33]. In our previous work, we further explored IFITM3 cholesterol interaction in the context of IFITM3 *S*-palmitoylation and antiviral activity [27]. We used photoaffinity cholesterol reporter and a chemo-proteomic platform to identify IFITM3 as a sterol-binding immune-associated protein in cells [27]. Photoaffinity cholesterol reporter can be crosslinked to interacting proteins using UV irradiation. Further overexpression studies and metabolic labeling with photoaffinity cholesterol reporter confirmed IFITM3–sterol interactions and identified the role of *S*-palmitoylation in mediating this interaction. A putative cholesterol binding motif CARC ^104^KCLNIWALIL^113^ N-terminal to the transmembrane domain was also identified, and it is conserved in great apes. NMR studies of recombinant IFITM3^89−133^ in membrane bicelles also confirmed structural perturbations in the presence of cholesterol. Many membrane proteins are known to have such a motif to mediate interactions with cholesterol [39,40]. Mutagenesis studies show that the loss of cholesterol binding does not impact *S*-palmitoylation or subcellular localization of IFITM3, but leads to a significant loss of resistance to influenza A virus and SARS-CoV-2 pseudovirus infection. Thus, IFITM3–cholesterol interactions might play an important role in blocking virus fusion and the release of genetic material in host cytosol during IAV and SARS-CoV-2 infection.

Using molecular dynamic studies of *S*-palmitoylated IFITM3 in a model membrane, we showed that there is increased interaction of the IFITM3 amphipathic helix with the membrane in the presence of cholesterol [27]. Rahman et al. showed that cholesterol’s binding potential in vitro is correlated with the membrane insertion depth of the amphipathic helix in silico [41]. Furthermore, recombinant IFITM3 binds to cholesterol in vitro. Loss-of-function mutations in IFITM3 disrupt the helical structure and reduce cholesterol binding.

All three interferon-induced human IFITM proteins have conserved Cys71, 72, and 105, as well as a conserved cholesterol binding motif. IFITM2 and IFITM3 protein sequence alignment shows 83% sequence identity. Interestingly, photoaffinity cholesterol reporter interacts with IFITM1 and IFITM3, but not with IFITM2 [27]. The *S*-palmitoylation levels of IFITM2 and IFITM3 correlated with cholesterol reporter labeling [27]. These results suggest that IFITM2 and IFITM3 interactions with cholesterol are determined by their *S*-palmitoylation levels. Evaluating their antiviral activity, IFITM3 exhibits greater antiviral activity against influenza virus and SARS-CoV-2 pseudovirus, whereas IFITM2 is more active against Ebola pseudovirus. Such differential activity of IFITM2 and IFITM3 against Ebola pseudovirus has also been seen in other studies [42]. These results suggest that differential interactions with cholesterol and *S*-palmitoylation impact the antiviral activity of IFITM proteins against different viruses. Thus, higher levels of *S*-palmitoylation enhance IFITM3’s interaction with cholesterol and inhibits viruses like IAV and SARS-CoV-2, whereas IFITM2 exhibits lower *S*-palmitoylation levels and cholesterol interactions but more effectively inhibits EBOV. Thus, differential *S*-palmitoylation levels and cholesterol interactions explain the different IFITM activities against viruses that enter through different endosomal compartments.

## 5. Mechanism of IFITM3’s Antiviral Activity and *S*-Palmitoylation Regulation

Many enveloped viruses, like the influenza A virus, use the endolysosomal pathway to enter cells. In the low pH of late endosomes, the viral fusion protein undergoes significant conformational changes to reveal a hydrophobic fusion loop which inserts into the target host membrane to initiate membrane fusion for pore formation. The fusion protein brings the two membranes together, overcoming the unfavorable electrostatic repulsion between the host and the viral membrane phospholipid headgroups and helps to create a hemifused stalk. This step is followed by an extension of the fusion diaphragm, and finally the membranes separate to form a fusion pore through which the virus’ genetic material escapes into the host cytosol [43]. Initially, it was suggested that IFITM3 blocks virus infection by disrupting cholesterol homeostasis in cells, leading to the accumulation of cholesterol in late endosomes and multivesicular bodies [36,37]. However, this is highly contentious, since other studies showed that cholesterol accumulation does not inhibit virus infection [38]. Using DiD-labeled viruses, it was shown that IFITM3 does not inhibit lipid mixing, but instead can inhibit content release in host cells. Recent live-cell imaging studies also show that IFITM3 colocalizes with virus particles and traffics to lysosomes, but does not inhibit membrane lipid mixing or hemifusion [28,29]. Despite its lack of effect on viral membrane hemifusion with endosomal membranes, IFITM3 could potentially block fusion pore formation and the release of viral genetic material in the cytosol [28,33]. It has been suggested that IFITM3 could modulate membrane curvature and stiffness to block fusion pore formation, a process that can be promoted by the presence of cholesterol in model membranes [20,33]. IFITMs have been shown to interact with cholesterol in cells, which is enhanced by *S*-palmitoylation [27,44]. Interestingly, molecular dynamic studies show that IFITM3 can cause local lipid sorting, leading to an increased concentration of lipids, disfavoring viral fusion at the hemifusion site and decreating the cholesterol concentration [44]. Further studies are needed to understand how IFITMs induce cholesterol and phospholipid sorting during hemifusion.

## 6. Conclusions

We discussed *S*-palmitoylation-regulated IFITM’s specificity and activity against viral infections in this review. Using chemical biology tools for metabolic labeling and live-cell imaging and the site-specific incorporation of lipid modification and photoaffinity cholesterol reporter, we have gained a better insight into IFITM’s antiviral activity, specificity, and regulation. There are important links between IFITM3’s site-specific modification and in-cell trafficking, membrane–lipid interactions, antiviral activity and specificity. These studies might enable the development of targeted antiviral approaches against emerging and re-emerging viral infections in the future.

We have described how use of chemical tools led to key insights into IFITM3’s mechanisms and regulation by *S*-palmitoylation. Metabolic labeling with palmitic acid reporter enabled the identification of IFITM3 *S*-palmitoylation and mutagenesis studies identified the role of this modification in IFITM3’s antiviral activity. Further Acyl-PEG exchange (APE) analysis identified sites and a number of modifications. The use of genetic code expansion and bio-orthogonal labeling techniques to perform live-cell trafficking studies of IFITM3 in infected cells showed that IFITM3’s antiviral activity and trafficking to incoming virus particles also requires *S*-palmitoylation at highly conserved Cys72, suggesting a role of site-specific fatty acylation. Moreover, site-specific lipidation enhanced IFITM3’s antiviral activity against the influenza A virus. Structural and biochemical studies with chemically lipidated recombinant IFITM3 showed that Cys72 palmitoylation can modulate IFITM3 conformation and interactions with membranes. Moreover, the use of photoaffinity cholesterol reporter suggests that cholesterol can directly interact with *S*-palmitoylated IFITMs in cells. Notably, S-palmitoylation levels regulate differential IFITM protein interactions with cholesterol in mammalian cells and the specificity of their antiviral activity towards different viruses. Collectively, these studies greatly enhanced our understanding of IFITM3’s biology and the role of *S*-palmitoylation, although there are still many outstanding questions on the IFITM family’s antiviral specificity, mechanism, and regulation.

Beyond antiviral immunity, IFITM3 has been shown to play a regulatory role in phosphoinositide 3-kinase (PI3K) signaling in B cells [45] and γ-secretase activity for amyloid-β production in neurons and astrocytes [46]. On the other hand, IFITMs are also present in placental cells and can cause pregnancy complications [47,48]. It will be very important to understand how *S*-palmitoylation regulates IFITM3 activity in these contexts. Interestingly, a recent study elucidated the role of IFITMs in the cellular uptake of molecules which break traditional drug design rules [49]. Understanding the role of IFITM3 and its regulation by *S*-palmitoylation can be set out a path for the cellular delivery of non-conventional drug molecules.

## Data Availability

Not applicable.
